# Supplementary material for: Comparative multiomics analysis of cell physiological state after culture in a basket bioreactor
Source: Sci Rep. 2022 Nov 23;12:20161. doi: 10.1038/s41598-022-24687-4 (PMC9686226; doi:10.1038/s41598-022-24687-4)
Supplement: Supplementary file 1 — Supplementary Information 1. [file 41598_2022_24687_MOESM1_ESM.zip › raw data/Transcriptomic raw data/6.Enrichment/1.GO/TE1vsTC1/GSEA/TC1/CATION_CHANNEL_COMPLEX/CATION_CHANNEL_COMPLEX(GO_0034703).html]

Details for gene set CATION\_CHANNEL\_COMPLEX(GO:0034703)[GSEA]


|  || Dataset | readcount.sample |
| Phenotype | sample.cls |
| Upregulated in class | TC1 |
| GeneSet | CATION\_CHANNEL\_COMPLEX(GO:0034703) |
| Enrichment Score (ES) | -0.1958192 |
| Normalized Enrichment Score (NES) | -1.0 |
| Nominal p-value | 0.0 |
| FDR q-value | 0.8967745 |
| FWER p-Value | 1.0 |
Table: GSEA Results Summary

  

Fig 1: Enrichment plot: CATION\_CHANNEL\_COMPLEX(GO:0034703)
  
Profile of the Running ES Score & Positions of GeneSet Members on the Rank Ordered List

  

| PROBE | DESCRIPTION   (from dataset) | GENE SYMBOL | GENE\_TITLE | RANK IN GENE LIST | RANK METRIC SCORE | RUNNING ES | CORE ENRICHMENT || 1 | 103217233 | SCN9A |  |  | 4384 | 1.474 | -0.0198 | Yes |
| 2 | 103238326 | SCN8A |  |  | 7215 | 1.204 | -0.0024 | Yes |
| 3 | 103244566 | KCNB1 |  |  | 10352 | 1.001 | -0.0229 | Yes |
| 4 | 103217215 | SCN3A |  |  | 10590 | 0.985 | 0.0841 | Yes |
| 5 | 103243548 | CACNB1 |  |  | 14442 | 0.743 | 0.0008 | Yes |
| 6 | 103217220 | SCN2A |  |  | 15754 | 0.658 | 0.0209 | Yes |
| 7 | 103238243 | CACNB3 |  |  | 17900 | 0.498 | -0.0154 | Yes |
| 8 | 103234628 | CATSPERG |  |  | 18560 | 0.434 | 0.0069 | Yes |
| 9 | 103221052 | SCN5A |  |  | 18678 | 0.424 | 0.0523 | Yes |
| 10 | 103237964 | CACNB2 |  |  | 18690 | 0.423 | 0.1024 | Yes |
| 11 | 103230887 | CATSPERE |  |  | 20018 | 0.270 | 0.0754 | Yes |
| 12 | 103243207 | SCN4A |  |  | 20096 | 0.259 | 0.1029 | Yes |
| 13 | 103217230 | SCN1A |  |  | 21086 | 0.000 | 0.0587 | No |
| 14 | 103221051 | SCN10A |  |  | 22400 | 0.000 | 0.0000 | No |
| 15 | 103221050 | SCN11A |  |  | 22401 | 0.000 | 0.0000 | No |
Table: GSEA details
[plain text format]

  

Fig 2: CATION\_CHANNEL\_COMPLEX(GO:0034703)
  
Blue-Pink O' Gram in the Space of the Analyzed GeneSet

  

Fig 3: CATION\_CHANNEL\_COMPLEX(GO:0034703): Random ES distribution
  
Gene set null distribution of ES for
 **CATION\_CHANNEL\_COMPLEX(GO:0034703)**
